# Virtual food exposure with positive mood induction or social support to reduce food anxiety in anorexia nervosa: A feasibility study

**DOI:** 10.1002/eat.24155

**Published:** 2024-02-17

**Authors:** Ludovica Natali, Valentina Meregalli, Katie Rowlands, Jerome Di Pietro, Janet Treasure, Enrico Collantoni, Paolo Meneguzzo, Elena Tenconi, Angela Favaro, Francesca Fontana, Enrico Ceccato, Alessandra Sala, Lucia Valmaggia, Valentina Cardi

**Affiliations:** ^1^ Department of General Psychology University of Padova Padova Italy; ^2^ Department of Neuroscience University of Padova Padova Italy; ^3^ Padova Neuroscience Center University of Padova Padova Italy; ^4^ Department of Psychological Medicine Institute of Psychiatry, Psychology & Neuroscience, King's College London London United Kingdom; ^5^ Department of Psychology Institute of Psychiatry, Psychology & Neuroscience, King's College London London United Kingdom; ^6^ Department of Psychiatry Eating Disorders Unit, Padova University Hospital Padova Italy; ^7^ Centro Provinciale di Treviso per i Disturbi del Comportamento Alimentare (Treviso Eating Disorders Unit) ULSS2 Marca Trevigiana Treviso Italy; ^8^ Centro Provinciale di Vicenza per i Disturbi del Comportamento Alimentare (Vicenza Eating Disorders Unit) ULSS8 Berica Vicenza Italy; ^9^ South London and Maudsley NHS Foundation Trust London United Kingdom; ^10^ Department of Psychiatry KU Leuven Leuven Belgium

**Keywords:** anxiety, avatar, eating disorders, exposure, learning, mood, social, support, virtual

## Abstract

**Objective:**

Aversive emotions toward food and the consequences of eating are at the core of anorexia nervosa. Exposure therapy is effective to reduce anxiety and avoidance toward feared stimuli. Based on the inhibitory learning framework, this study examined the feasibility to induce social support or positive mood to enhance the impact of a single session virtual food exposure on food‐related anxiety in anorexia nervosa.

**Method:**

One hundred and forty‐five patients were randomized to: (1) virtual food exposure (i.e., baseline condition), (2) virtual food exposure plus positive mood induction (i.e., positive mood condition), or (3) virtual food exposure plus social support (i.e., social support condition). They completed self‐report assessments of anxiety toward virtual foods, general anxiety, positive mood, social support, and hunger, before and after virtual food exposure. Number of eye gazes and touches toward foods were recorded during the virtual reality exposure.

**Results:**

Patients had lower levels of anxiety toward virtual foods in the positive mood condition, compared to the baseline condition [*F*_(2,141)_ = 4.36, *p* = .015; medium effect size]. They also touched food items more often in the baseline condition. No other significant changes were found.

**Discussion:**

Virtual food exposure enhanced by positive mood induction seems a feasible approach to strengthen the impact of food exposure in anorexia nervosa.

**Public Significance:**

This research contributes to the understanding of how patients with anorexia nervosa can be supported to overcome fear and anxiety around food. Virtual reality enables patients to expose themselves to difficult situations (e.g., kitchen with foods of various calorie contents) while experiencing positive stimuli, such as a loving and kind pet or a supportive avatar.

## 1 INTRODUCTION

Anorexia nervosa is a psychiatric disorder prototypically marked by anxiety around eating and food avoidance (American Psychiatric Association, 2013; Levinson & Byrne, 2015; Steinglass et al., 2010). This implies that the primary goal of treatment is nutritional rehabilitation, which consists in restoring weight loss, together with healthier and more flexible eating patterns. However, the conundrum of anorexia nervosa's treatment is that nutritional rehabilitation does not seem to work satisfactorily and even when weight is restored, most patients continue to struggle with negative emotions around food, rigid eating habits, and avoidance of specific nutrients (e.g., Levinson & Byrne, 2015; Murray et al., 2019; Schebendach et al., 2008).

Behavioral theory posits that repeated exposure to a feared stimulus is key to “habituate” to the stimulus and to overcome the fear, anxiety, and avoidance associated with it (i.e., this is defined as “the habituation model of extinction”). In anorexia nervosa, this process alone seems to fail, as repeated exposure to food in treatment settings often reinforces, and does not weaken, food aversion (e.g., Treasure et al., 2012). In recent years, the proposal that habituation is the main mechanism of fear extinction has been questioned and a new framework has been developed, the “inhibitory learning model” (Craske et al., 2014). This model proposes that repeated exposure to the feared stimulus in the absence of the aversive feared consequences (e.g., repeated exposure to food in the absence of loss of control, also defined as “expectancy violation”), leads to the development of a new and safer memory, *together with* the weakening (rather than the cancellation) of the older, “negative” memory (Craske et al., 2014). Repeated practice reinforces the new memory but does not “erase” the negative one. The coexistence of the positive and negative memories means that fear and anxiety are likely to return. To prevent this from happening and to reinforce new learning while inhibiting activation of the older memory trace, several strategies have been proposed, both in relation to anxiety disorders (Murray et al., 2019) and eating disorders (Melles & Jansen, 2023; Murray et al., 2016; Reilly et al., 2019). These strategies include, for example, variability in the types of stimuli presented, affect labelling, and emotion regulation during exposure (Craske et al., 2018; Craske et al., 2014; Melles & Jansen, 2023; Murray et al., 2016; Reilly et al., 2019). The hypothesis that these strategies can enhance extinction learning of the fear memory and new learning of safer memories remain to be tested, together with the proposal that food exposure enhanced by any of these strategies would be a feasible approach for patients.

Virtual reality (VR) provides an engaging setting which might help testing exposure‐based techniques (Valmaggia, 2017). It enables exposure to a variety of stimuli at the same time, while keeping exposure conditions constant across individuals. It also enables the manipulation of specific variables during exposure (e.g., through the insertion of specific elements in the virtual environment), and the measurement of their effect on participants' behavior in real time. Importantly, VR allows to collect “passive” measurements which would be very difficult to collect during “in vivo” exposure. These are, for example, food‐related eye gaze and approach behavior (through recording the number of times a participant looks at a food item or holds a food item, respectively). In eating disorders, VR has been used mostly for food cue exposure in patients with binge eating episodes and also to evaluate and modify body distortions and body image (Riva et al., 2021; Wiebe et al., 2022). VR has been employed less often in the context of anorexia nervosa, where preliminary findings indicate that this technology is helpful to probe food‐related fear and to some extent also modify eating‐related behavior (Cardi, Krug, et al., 2012; Gorini et al., 2010).

In this study, the feasibility of using VR food exposure alone or in combination with positive mood induction or social support was tested. In addition, it was examined whether positive mood induction and/or social support would induce a greater reduction of food‐related fear and anxiety, over and above VR food exposure alone. These conditions were aimed at strengthening the development of a “safer” memory during food exposure. The first condition involved the presence of a virtual pet, with the intent of inducing positive mood. The second condition incorporated an avatar to provide motivational enhancement through social support. Previous research in animals (Ferreira et al., 2019) and nonclinical populations (Zbozinek et al., 2015) generally supports the idea that positive mood and social support can enhance new (safer) learning. In the context of anorexia nervosa, studies have shown that both positive mood induction and motivational enhancement from peers are associated with increased calorie intake during a standard test meal and decreased attention bias to food after the test meal compared to a control condition (Cardi et al., 2015; Cardi, Kan, et al., 2012; Cardi et al., 2013). Additionally, it has been demonstrated that encouragement from people with lived experience is highly endorsed by patients over time and linked to a decrease in eating disorder symptoms (Cardi et al., 2013).

Based on these premises, the aim of this study was to test whether VR food exposure with positive mood induction or social support would be feasible and associated with a greater reduction of virtual food‐related anxiety compared to VR food exposure alone in anorexia nervosa. Self‐reported general anxiety, perceived positive affect, social support, hunger levels, and food‐related anxiety were measured before and after exposure. Number of touches and gazes directed toward virtual foods were measured during each exposure session by the VR headset. The main hypothesis was that the positive mood and social support conditions would be feasible and associated with lower levels of food‐related anxiety after exposure compared to the virtual kitchen alone condition. The secondary hypotheses were that perceived positive affect would be highest after exposure to the positive mood condition and that perceived social support would be highest after exposure to the social support condition. No specific hypotheses about food touches and gazes were formulated, based on the exploratory nature of the study.

## 2 METHOD

### 2.1 Participants

Participants were recruited from outpatient or daycare eating disorder centers in Italy (north‐east) or the UK (London). Inclusion criteria were: (1) fluency in Italian or English, (2) attending current outpatient or daycare treatment for anorexia nervosa (diagnosis at the start of treatment), and (3) a Global Score of the Eating Disorder Examination Questionnaire (EDE‐Q) above cut‐off (EDE‐Q Global Score ≥2.8; Mond et al., 2008). Exclusion criteria were: (1) age under 14 years, (2) body mass index (BMI, kg/m^2^) in the overweight or obesity range (BMI > 25), (3) self‐reported diagnosis of neurological disorders, (4) self‐reported diagnosis of psychosis or substance abuse disorders, (5) visual/hearing impairments not corrected by glasses/ear implants, and (6) non‐tolerance of exposure to VR (i.e., cybersickness, dizziness).

Ethical approval was obtained by the ethical committees of the University of Padova (reference number: 4293) and hospital of Vicenza (reference number: 1831) in Italy, and by the Research Ethics Committee North West – Liverpool East (reference number: 18/NW/0853) in the UK. The procedure was conducted in accordance with the latest version of the Declaration of Helsinki. Written informed consent was provided by all participants or by their parents or legal guardian.

An a‐priori power calculation was conducted in G*Power 3 (Faul et al., 2007) based on a repeated measures analysis of variance to compare the three different groups of participants (each group allocated to a different experimental condition), before and after exposure, with an expected medium‐sized change in food‐related anxiety (Cardi et al., 2015; Cardi et al., 2019; Cardi et al., 2013; Steinglass et al., 2014). The power calculation indicated that a total sample of 117 participants in total would be sufficient to detect a medium effect size (*f* = 0.25) with 95% of power and a significance level of 5%.

### 2.2 Materials

#### 2.2.1 VR environment

The VR kitchen environment was developed by the KCL VR Research Lab in the Unity3D game engine, using the Oculus Integration SDK for Unity, and installed on Oculus Quest 2 headsets. Each headset had six degrees of freedom (6DoF) technology, allowing for continuous rotational and positional tracking. The screen had a resolution of 1832 × 1920 pixels per eye. Patients were fully immersed in a room‐scale experience, with two Oculus Touch controllers enabling with User Interface (UI) menus and scene objects.

**FIGURE 1:**
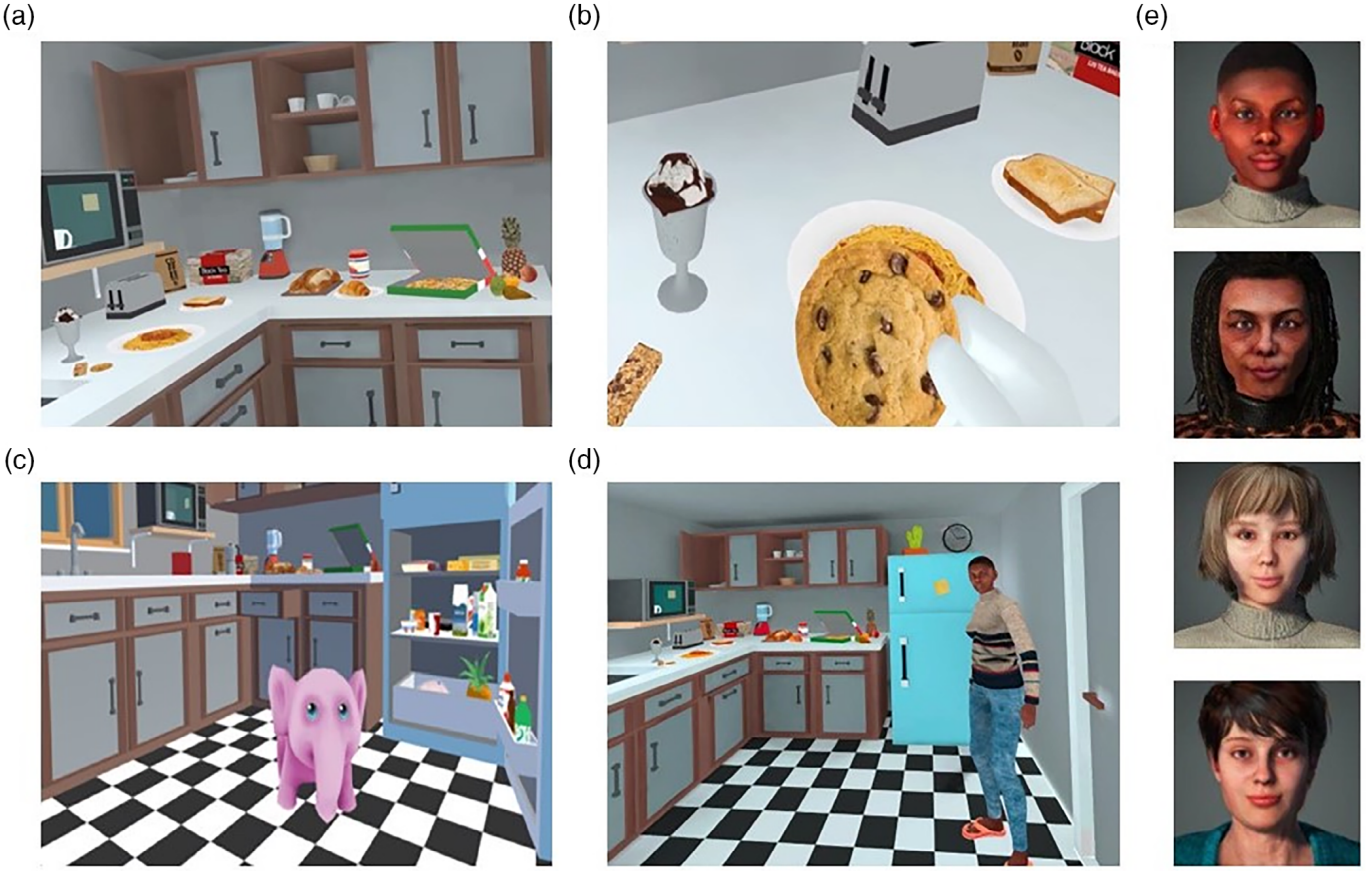
(a) Virtual kitchen across conditions. (b) Participant handling a food item in the virtual kitchen. (c) Pet in the positive mood induction condition. (d) Selection of avatars available to participants. (e) Young black female avatar standing in the kitchen and talking during the virtual reality exposure.

The VR scenario consisted of a kitchen which stored foods of different calorie content (see Figure 1a,b; a full list of the virtual foods is provided in Table 1). Foods were selected from those listed and rated (i.e., based on the level of anxiety they elicited) by patients with anorexia nervosa who participated in a study on in vivo exposure to food (Cardi et al., 2019). Once in the kitchen, patients could freely move, open the cupboards and the fridge, and grab and hold the foods. They could also use joystick‐driven smooth locomotion and teleportation, as an alternative locomotion to explore and interact with the environment. Three different versions (i.e., conditions) of the same virtual kitchen scenario were used: virtual kitchen alone (i.e., baseline condition), virtual kitchen plus a virtual pet to induce positive mood (i.e., positive mood condition), and a virtual kitchen plus an avatar to induce social support (i.e., social support condition). In the positive mood condition, participants explored the kitchen accompanied by a virtual pet, a pink elephant making soft gurgling noises when touched, and following the participant in the environment (see Figure 1c). In the social support condition, patients were accompanied in the kitchen by a virtual avatar (they could choose between four different avatars; Figure 1d,e). During exposure to the virtual kitchen, the avatar spoke a supportive and motivational script, encouraging participants to challenge the eating disorder voice (see Table S1). The interactions that participants could have with the VR environment consisted in moving around, opening cupboards, fridge and freezer, touching and holding foods, and touching the pet.

**TABLE 1:** List of virtual foods in the kitchen.

Apple Black tea Lager beer Bread Toast bread Chocolate chip cookie Chocolate granola bar Coffee beans Croissant Draft beer Egg sandwich Crunch crisps Chicken nuggets Chocolate ice cream Chocolate chip ice cream Frozen chips Low fat strawberry yoghurt	Orange Pear Pineapple Canned baked beans in tomato sauce Canned baked beans Canned garden peas Canned tinned tomato Canned tuna chunks Fusilli dried pasta Whole penne pasta Vanilla filled cake Cheese Crème Fraiche Full‐fat mayo Packed apple juice Large eggs Lightly salted butter Low fat milk	Pizza Raspberry Siracha Spaghetti Bolognese Sushi plate with soy White toast Canned chicken soup Cup noodle Macaroni and cheese Wheat flour Chocolate syrup Whipped cream Lemon sparkly drink Tomato ketchup Yellow mustard Raw sushi rolls Red bell pepper Carrot Whole milk	Pirate charms Summer berry crisp Wheat crunchies Original chips Cane sugar Cookie crumby Salt crackers Bubble gum mega big Dark chocolate Lollipops Broccoli Cabbage Red bell pepper Coffee Fried eggs with bacon Green soup Pepperoni Peanut butter Fish fingers

#### 2.2.2 General assessment

Patients answered a demographic questionnaire including questions on age, nationality, years of education, and illness duration. Patients also completed the following self‐report questionnaires.

*Eating Disorder Examination Questionnaire (EDE‐Q)* (Calugi et al., 2017; Fairburn & Beglin, 1994). The EDE‐Q is a 28‐item questionnaire assessing eating disorder psychopathology. It provides four subscale scores (Restraint, Eating Concern, Shape Concern, and Weight Concern) and a global score. Scores range from 0 to 6, and higher scores indicate greater severity. The Cronbach's alpha of the global score in this study was 0.9.

*Depression and Anxiety Stress Scales (DASS‐21)* (Bottesi et al., 2015; Lovibond & Lovibond, 1995). The DASS‐21 is a 21‐item self‐report measure. The scale comprises three subscales to measure symptoms of depression, anxiety, and stress. Higher scores indicate greater symptom severity. The Cronbach's alpha for the Depression and Anxiety subscales was 0.8. The Cronbach's alpha of the Stress scale was 0.9.

#### 2.2.3 Pre‐ and post‐virtual reality exposure measures

In the virtual environment, before and after exposure to the kitchen, patients were asked questions regarding anxiety level (i.e., “How anxious do you feel right now?”), hunger (i.e., “How hungry do you feel right now?”), positive mood (i.e., “How happy do you feel right now?”), perceived social support (i.e., “How much do you feel supported by others right now?”), and food‐related anxiety (i.e., “Imagine eating this food now, how anxious do you feel?”), on a visual analogue scale ranging from 0 to 100. Food‐related anxiety (scale 0–100) was measured in response to viewing six virtual foods before and after exposure to the kitchen. The order of appearance of the food items was randomized. The six foods used before exposure (i.e., apple, banana, sandwich, crisps, chocolate biscuits, slice of pizza) were matched to those presented post exposure (i.e., orange, avocado, pasta with vegetables, fries, chocolate cake, ice cream) in terms of overall calorie content. The resulting matching pairs were apple‐orange; banana‐avocado; crips‐fries; sandwich‐pasta with vegetables; chocolate biscuits‐ice cream; chocolate cake‐slice of pizza. These have been listed from the “least threatening” to the “most threatening,” based on the food choices that patients had made for themselves in the in‐vivo food exposure study published in 2019 (Cardi et al., 2019).

#### 2.2.4 Other VR measures

During exposure, the VR headset automatically recorded the number of eye‐gazes and touches directed toward the foods stored in the kitchen.

#### 2.2.5 Co‐development of the VR environments with people with lived experience

The VR environments were co‐developed with input from people with lived experience of the illness. All participants involved in this advisory phase did not take part in the study and therefore their data are not included. In order to minimize burden on this small group of volunteers, they were not asked to complete questionnaires or provide data in any other way. People with lived experience of eating disorders were first invited to participate to a focus group to discuss strategies for positive mood induction. The focus group included 10 adult participants (above 18 years old), who identified themselves as currently suffering from an eating disorder (which they did not have to describe to others in the group). Two were males. They were asked to discuss strategies to increase positive mood or relax that they would normally use in their own time. Participants came up with three main strategies: listening to favorite music, watering plants, and cuddling a pet. The latter was the most endorsed option. Participants were then asked to have a conversation around the characteristics of the pet. They described pros and cons of having a “real‐looking” or “fantasy‐pet” in the VR kitchen. They all agreed that having a fantasy pet, something which they could never find in real life, would have been the best option to experience feelings of openness and positive affect. One participant proposed then a pink elephant and all endorsed this proposal. The selection of a pet to induce positive mood aligns to previous studies demonstrating that exposure to images of small animals is associated with increased positive mood in patients with eating disorders (Cardi et al., 2015). This is consistent with findings from other clinical populations too (e.g., patients with depression, schizophrenia, dementia), where interacting with real or virtual/robot‐like pets has been associated with mood improvement (Moyle et al., 2013; Virtues‐Ortega et al., 2006).

Following the focus group and the development of the VR kitchen scenario, eight adult individuals (1 male, 7 females, all above 18 years old and all identifying themselves as currently suffering from an eating disorder) were invited to the lab to try the VR kitchen. They participated individually and again no data were collected to reduce burden. The principal investigator asked each participant to describe their experience of the kitchen and the virtual foods. All participants agreed that exposure to the kitchen created some level of “tolerable” distress. All found the kitchen pleasant in terms of brightness and colors. They all liked the variety of foods and found the food objects realistic (although not “too realistic,” which helped them to approach the items). Participants were also exposed to the positive mood condition and asked to use one word to describe the main characteristic of the pet. Three patients commented that it was “soothing,” one that it was “comforting,” two that it was “sweet,” one that it was “cheerful,” and one that it was “funny.”

Finally, the script spoken by the avatar (see Table S1) was derived from a library of recovery narratives developed and tested in previous studies (Cardi, Kan, et al., 2012; Cardi et al., 2013) and was revised by three people with lived experience of the illness, who provided written feedback by email to the principal investigator.

### 2.3 Procedure

**FIGURE 2:**
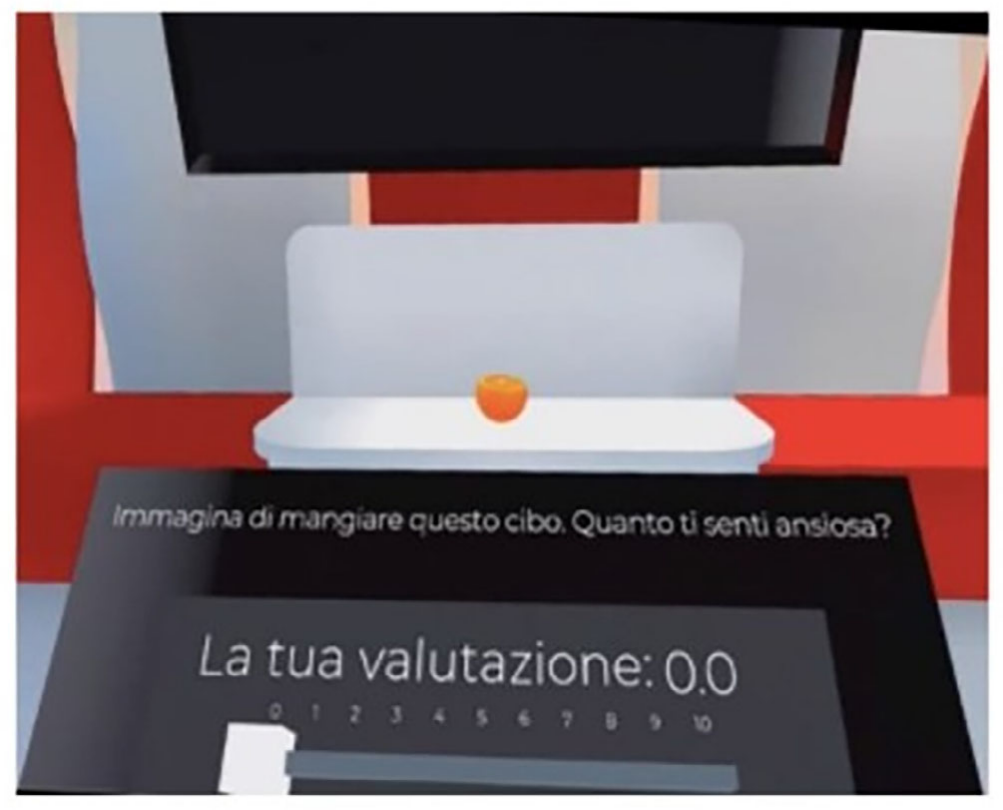
The podium that patients used to answer questions in the virtual reality environment.

On arrival at the laboratory, patients completed an online survey via Qualtrics (https://www.qualtrics.com) including the demographic questionnaire, the Eating Disorder Examination Questionnaire (Calugi et al., 2017; Fairburn & Beglin, 1994), and the Depression and Anxiety Stress Scales (Bottesi et al., 2015; Lovibond & Lovibond, 1995). Participants' weight and height were obtained from medical records (for recruitment in Italy) or self‐reported (for recruitment in the UK), and used to calculate the BMI (weight (kg)/[height (m)]^2^). Participants were then guided to familiarize themselves with the VR headset and instructed to start the VR kitchen application. Once the application was launched, patients were redirected in a colorful room with a screen displaying instructions and a podium where they answered questions using visual analogue scales (see Figure 2). In this room, patients completed the pre‐VR exposure measures. Patients were then randomized by the VR headset to one of the three conditions. They were given the instruction to explore the environment as much as they felt comfortable with by a pre‐recorded voice. After the exposure to the kitchen, patients were once again automatically directed to the colorful room to complete the post‐VR measures.

The experimenter remained in the same room as the participant for the entire duration of the procedure. Each participant was exposed to one condition and one VR session only. The entire session lasted approximately 8–10 minutes (including exposure to the kitchen and completion of pre‐ and post‐VR assessments). Exposure to the VR kitchen did not exceed 5 min, the shortest duration needed to explore all elements of the kitchen. Considering that this study was aimed at testing the feasibility of exposure to the VR environment, the research team took a conservative approach with the plan to slowly build on preliminary findings of acceptability and participants' feedback to extend the duration of exposure in multi‐session, future treatment studies.

### 2.4 Statistical analyses

Data were analyzed using the software R studio (R Core Team, 2023; version 4.2.2). The outcome variables were self‐reported anxiety, hunger, perceived social support, positive mood, and food‐related anxiety. Food‐related anxiety was computed as the mean of the ratings provided in response to the virtual food items. The independent variables were time (i.e., pre‐ and post‐VR exposure) and condition (i.e., baseline condition, positive mood condition, social support condition). Given differences between conditions in some of the baseline ratings, analyses of covariance (ANCOVA) were computed to assess differences between conditions for anxiety toward virtual foods, general anxiety, hunger, positive affect, and perceived support following VR exposure, adjusting for baseline ratings (Wan, 2021).

(1) ∼Baseline×VR condition

ANCOVA models were performed using the “lm” function (stats package, R Core Team, 2021) and explored with the “anova_test” function (rstatix package; Kassambara, 2023). Post hoc tests were computed using the “emmeans_test” function (rstatix package; Kassambara, 2023). Bonferroni correction was used for multiple comparisons. Analyses of variance (ANOVA; “aov” function, stats package, R Core Team, 2023) were performed to investigate differences between conditions for number of touches and gazes directed toward food items. Post hoc comparisons were performed with the Tukey's test (“TuckeyHSD” function, stats package, R Core Team, 2023). A posteriori paired sample *t*‐tests were conducted to check for significant changes in food‐related anxiety in each condition. Cohen's *d* was calculated (effectsize package, R Core Team, 2023) to estimate effect sizes. Cohen's *d* was described as negligible (=0 and <0.15), small (≥0.15 and <0.40), medium (≥0.40 and <0.75), large (≥0.75 and <1.10), very large (≥1.10 and 1.45), and huge (>1.45) (Cohen, 1992).

Finally, a posteriori Spearman's rho correlational analysis was conducted to examine whether changes in the level of food‐related anxiety (i.e., pre‐VR food‐related anxiety ratings – post‐VR food‐related anxiety ratings) were associated with the degree of interaction with the virtual environment (i.e., number of touches and gazes toward food items).

## 3 RESULTS

### 3.1 Demographic and clinical characteristics

**FIGURE 3:**
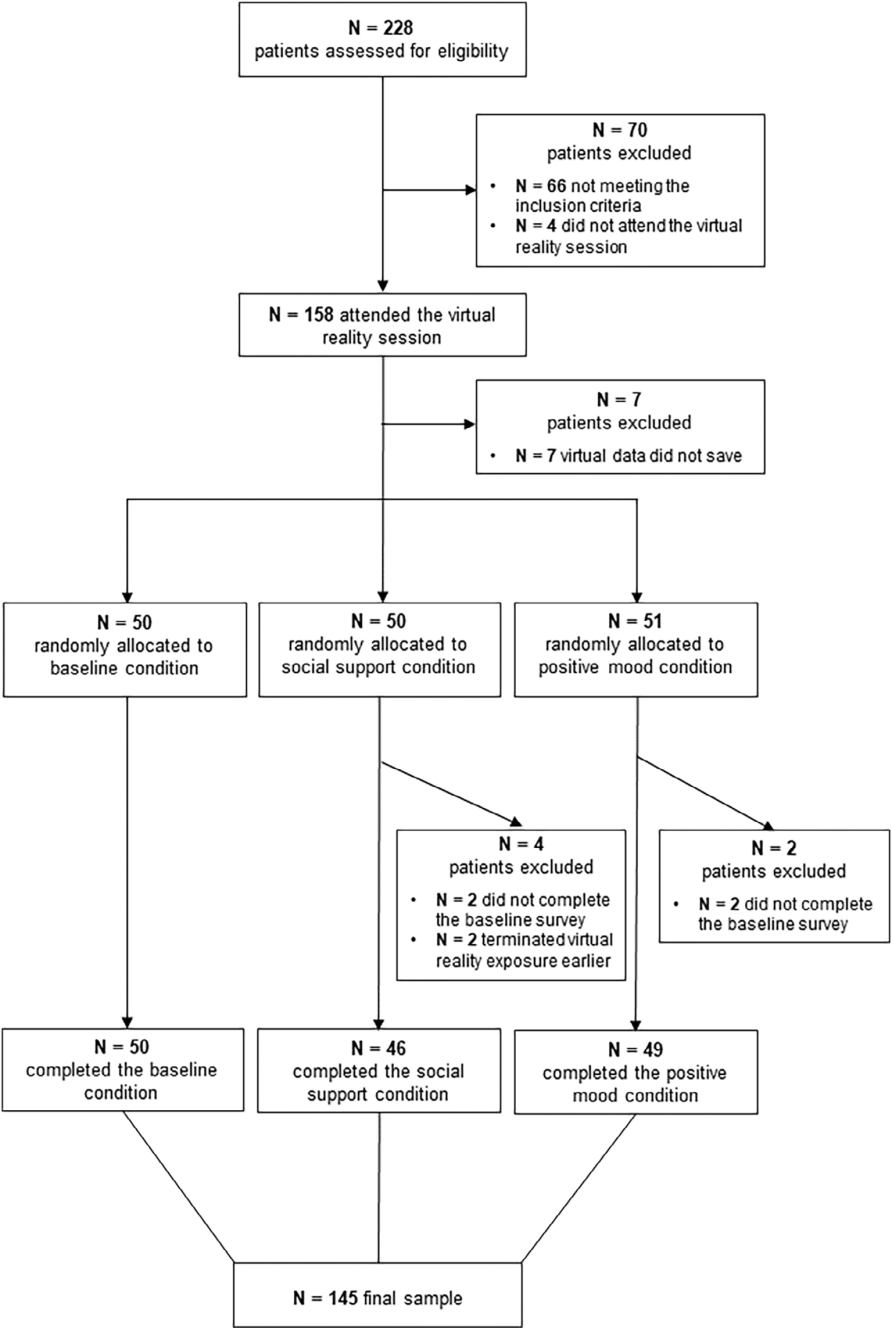
A CONSORT diagram describing the flow of participation to the study.

Two hundred and twenty‐eight female patients expressed interest in participating and were assessed for eligibility. Sixty‐six patients did not meet the inclusion criteria: two patients reported a BMI in the overweight or obesity range, two patients reported a comorbid disorder which was among the exclusion criteria (psychosis or substance abuse disorders), one patient was younger than 14, and 61 patients had an EDE‐Q Global Score below cut‐off. Seventeen patients did not complete the procedure: four patients did not complete the baseline survey, for seven patients VR data did not save, four patients did not attend the VR session, and two patients terminated the VR exposure earlier (one patient was afraid of falling due to osteoporosis and one patient found the exposure to virtual foods too stressful). The final sample included 145 patients. The flow of participation in the study is described in Figure 3.

Participants' sociodemographic and clinical variables are reported in Table 2. Descriptive and clinical data for each group separately are reported in Table S1. On average, patients reported clinically significant levels of eating disorder psychopathology (EDE‐Q; Mond et al., 2008) and severe levels of psychological distress (DASS‐21; Lovibond & Lovibond, 1995). Fifty patients (34.48%) were assigned to the baseline condition, 46 patients were assigned to the social support condition (31.72%), and 49 patients were assigned to the positive mood condition (33.79%). Among those who were assigned to the social support condition, 17 selected the older white female avatar (36.96%), 13 chose the younger white female avatar (28.26%), 13 the younger black female avatar (28.26%), and 2 the older black female avatar (4.35%).

**TABLE 2:** Participants' demographic and clinical variables expressed as means (standard deviations) or frequencies (%).

Variables	*N*	*M* (*SD*) or frequency (%)
Age	144	21.75 (6.68) [min: 14.00; max: 50.00]
Gender	145	
Female		140 (96.55%)
Male		2 (1.38%)
Non‐binary		2 (1.38%)
Other		1 (0.69%)
Site of data collection	145	
Italy		91 (62.76%)
UK		54 (37.24%)
Ethnicity	144	
White ethnicity		136 (94.44%)
Other ethnicity		8 (5.55%)
Illness duration (years)	142	5.47 (5.68) [min: 0.33; max: 26.00]
Years of education	145	13.43 (3.31)
Body mass index (kg/m^2^; >18 years old)	87	17.00 (2.56)
Percent median BMI (<18 years old)	41	83.21 (8.22)
Binge eating (yes vs. no)^a^	145	23 (15.86%)
Purging (yes vs. no)^b^	145	28 (19.31%)
EDE‐Q Global Score	145	4.29 (0.77)
EDE‐Q Restraint	145	4.03 (1.19)
EDE‐Q Eating Concern	145	3.47 (1.06)
EDE‐Q Weight Concern	145	4.60 (1.06)
EDE‐Q Shape Concern	145	5.04 (0.82)
DASS total score	145	68.72 (23.39)

*Note*: Sociodemographic and clinical variables expressed as mean (standard deviation) and frequency (percentage).

Abbreviations: BMI, body mass index; DASS, Depression and Anxiety Stress Scales; EDE‐Q, Eating Disorder Examination Questionnaire.

^a^ “Binge eating” is a categorical variable: a “yes” was given when participants reported at least four binge episodes in the last month.

^b^ “Purging” is a categorical variable: a “yes” was given when participants reported at least one episode in the last month.

### 3.2 Differences between VR conditions in food‐related anxiety

**FIGURE 4:**
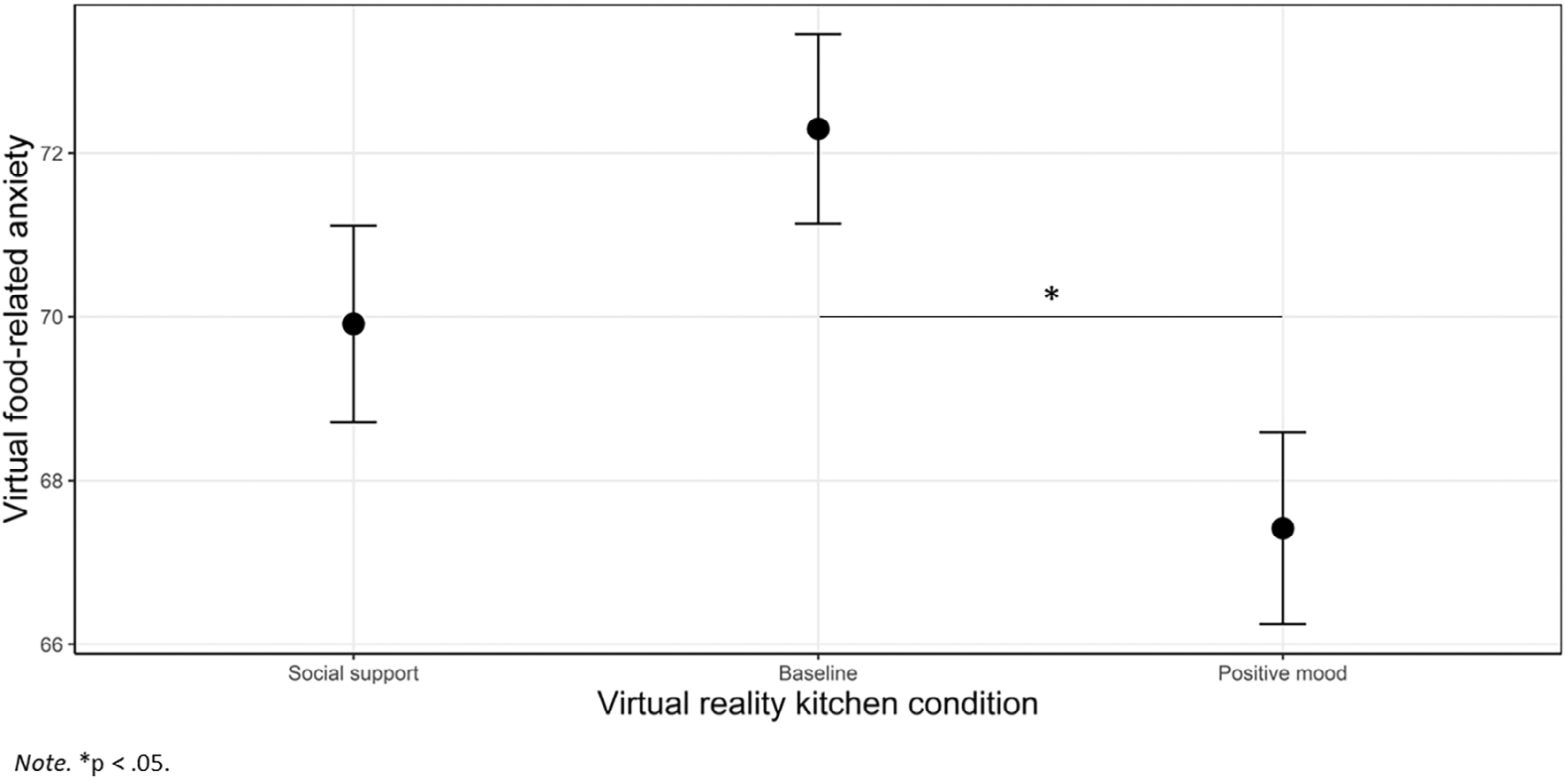
Differences between conditions in food‐related anxiety post‐exposure, adjusted for baseline measures.

In all the ANCOVA models, pre‐exposure ratings were significantly related to post‐exposure ratings (*p* < .001). After controlling for baseline ratings, there was a significant effect of Condition on virtual food‐related anxiety (*F*_(2,141)_ = 4.36, *p* = .015, $\eta_{p}^{2}=0.06;$ Figure 4): participants in the positive mood condition reported lower levels of virtual food‐related anxiety after exposure compared to baseline, with a medium effect size (*p* = .011; social support condition vs. baseline condition: *p* = .466; social support condition vs. positive mood condition: *p* = .417). Self‐reported food‐related anxiety ratings before and after exposure to each condition are reported in Table 3 and pairwise comparisons are displayed in Table 4.

**TABLE 3:** Self‐reported ratings before and after exposure to each virtual reality condition.

Variables	Virtual kitchen (*n* = 50)		Virtual kitchen + social support (*n* = 46)		Virtual kitchen + positive mood (*n* = 49)	
	Pre, *M* (*SD*)	Post, *M* (*SD*)	Pre, *M* (*SD*)	Post, *M* (*SD*)	Pre, *M* (*SD*)	Post, *M* (*SD*)
Virtual food‐related anxiety^a^	67.70 (11.83)	70.78 (12.42)	69.20 (14.95)	69.66 (15.76)	71.66 (10.90)	69.21 (11.65)
Perceived support	54.66 (23.70)	56.14 (25.86)	59.22 (27.41)	60.22 (28.76)	61.06 (20.78)	61.37 (23.78)
Positive mood	42.30 (20.25)	44.18 (22.80)	50.83 (27.65)	49.74 (28.86)	57.25 (26.42)	55.49 (26.61)
Hunger	24.26 (27.23)	27.96 (30.83)	15.33 (24.58)	19.61 (25.74)	27.71 (29.78)	29.96 (32.73)
General anxiety	63.14 (22.47)	59.92 (24.40)	51.28 (29.97)	56.57 (28.98)	57.39 (25.68)	59.53 (24.00)
Food‐related touches	20.44 (9.06)		12.41 (8.59)		16.02 (8.27)	
Food‐related gazes	367.52 (143.26) [min: 72.00–max: 929.00]		330.00 (97.89) [min: 149.00–max: 589.00]		326.04 (137.16) [min: 5.00–max: 969.00]	

*Note*: Data expressed as means and standard deviations. *N* = 143. Variables were rated on a visual analogue scale from 0 (i.e., not at all) to 100 (i.e., extremely).

^a^ “Virtual food‐related anxiety” was computed as mean of the food‐related anxiety ratings.

**TABLE 4:** Pairwise comparisons, from the analysis of covariance and analysis of variance models.

Dependent variable	Condition 1	Condition 2	Mean difference	*SE*	*t*	Cohen's *d*	95% CI		*p*
							Lower	Upper	
Virtual food‐related anxiety^a^	Social support	Baseline	−2.832	1.668	−1.428	−0.292	−0.789	0.205	.466
		Positive mood	2.499	1.679	1.488	0.306	−0.194	0.807	.417
	Baseline	Positive mood	4.882	1.653	2.953	0.599	0.100	1.097	.011
Perceived support	Social support	Baseline	−0.584	1.834	−0.318	−0.065	−0.562	0.431	1.000
		Positive mood	0.736	1.838	0.400	0.082	−0.416	0.580	1.000
	Baseline	Positive mood	1.319	1.810	0.729	0.147	−0.343	0.638	1.000
Positive mood	Social support	Baseline	−2.583	2.244	−1.151	−0.237	−0.739	0.264	.755
		Positive mood	0.379	2.246	0.169	0.035	−0.465	0.535	1.000
	Baseline	Positive mood	2.963	2.254	1.314	0.272	−0.231	0.776	.573
Hunger	Social support	Baseline	0.545	2.609	0.209	0.043	−0.456	0.543	1.000
		Positive mood	1.986	2.643	0.751	0.157	−0.349	0.663	1.000
	Baseline	Positive mood	1.441	2.548	0.565	0.114	−0.374	0.602	1.000
General anxiety	Social support	Baseline	5.066	3.746	1.352	0.281	−0.224	0.786	.535
		Positive mood	1.370	3.717	0.369	0.076	−0.424	0.576	1.000
	Baseline	Positive mood	−3.696	3.638	−1.016	−0.205	−0.695	0.285	.934
Food‐related touches^b^	Social support	Baseline	−8.027	1.767	−4.543	−0.928	−1.441	−0.416	<.001
		Positive mood	−3.607	1.775	−2.032	−0.417	−0.918	0.084	.108
	Baseline	Positive mood	4.420	1.738	2.542	0.511	0.019	1.004	.032
Food‐related gazes^b^	Social support	Baseline	−37.520	26.226	−1.431	−0.292	−0.789	0.204	.328
		Positive mood	3.959	26.354	0.150	0.031	−0.467	0.528	.988
	Baseline	Positive mood	41.479	25.804	1.607	0.323	−0.166	0.812	.246

*Note*: *N* = 143. Variables were rated on a visual analogue scale from 0 (i.e., not at all) to 100 (i.e., extremely).

^a^ “Virtual food‐related anxiety” was computed as mean of the food‐related anxiety ratings.

^b^ Pairwise comparisons referred to analysis of variance.

### 3.3 Differences between VR conditions in general anxiety, positive mood, perceived support, and hunger

No between‐condition differences were found with regards to general anxiety (*F*_(2,141)_ = 1.00, *p* = .372, $\eta_{p}^{2}=0.01$), positive mood (*F*_(2,141)_ = 1.03, *p* = .361, $\eta_{p}^{2}=0.01$), perceived support (*F*_(2,141)_ = 0.27, *p* = .766, $\eta_{p}^{2}=0.004$), or hunger (*F*_(2,141)_ = 0.31, *p* = .736, $\eta_{p}^{2}=0.004$). Self‐reported ratings before and after exposure to each condition are reported in Table 3 and pairwise comparisons are displayed in Table 4.

### 3.4 Changes across time in food‐related anxiety

After VR exposure, food‐related anxiety was significantly increased in the baseline condition, with a small effect size (*t*(49) = −2.47, *p* = .017, *d* = −0.35), and significantly decreased in the positive mood condition, with a small effect size (*t*(48) = 2.34, *p* = .024, *d* = 0.33). No significant changes in food‐related anxiety were found in the social support condition (*t*(45) = −0.34, *p* = .734, *d* = −0.05).

### 3.5 Differences between VR conditions in number of food gazes and touches

**FIGURE 5:**
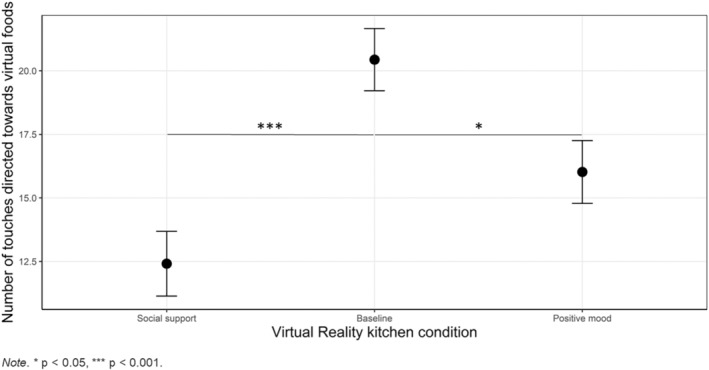
Differences between conditions in total number of food touches.

No significant differences between conditions were found in total number of gazes toward virtual foods (*F*_(2,142)_ = 1.57, *p* = .212, $\eta_{p}^{2}=0.02$). A significant effect of Condition was found for food touches (*F*_(2,142)_ = 10.39, *p* < .001, $\eta_{p}^{2}=0.13$; Figure 5). Post hoc tests showed that patients in the baseline condition touched the virtual foods more often than patients in the social support condition, with a large effect size (*t* = 4.54, *p* < .001, *d* = 0.93), and more than patients in the positive mood condition, with a medium effect size (*t* = 2.54, *p* = .032, *d* = 0.51). No significant differences were found between the positive mood condition and the social support condition in total number of touches (*p* = .108). A full report on pairwise comparisons is displayed in Table 4.

### 3.6 Relationship between food touches and gazes and changes in food‐related anxiety

Correlational analyses did not show any significant correlation between changes in food‐related anxiety and total number of touches (*ρ* = −0.07, *p* = .379) or number of gazes directed toward food items (*ρ* = −0.15, *p* = .072).

## 4 DISCUSSION

This study tested the feasibility of using different psychological strategies to enhance inhibitory learning of food‐related anxiety in patients with anorexia nervosa. A VR kitchen was compared to a VR kitchen incorporating positive mood induction or social support in reducing anxiety related to virtual foods. One hundred and fifty‐eight patients with anorexia nervosa were exposed to the VR environment and only two interrupted the exposure prematurely (1.2%). This shows the overall feasibility of the approach. The positive mood induction condition was associated with greater reduction in food‐related anxiety compared to the other two conditions, which suggests that strategies to improve positive mood might be helpful in inhibiting food‐related anxiety. Learning mechanisms play a crucial role in the development of food‐related fears and anxiety in anorexia nervosa (Cardi et al., 2019) and effective inhibitory learning of food fears require developing new positive associations with food stimuli. Patient‐centered approaches to induce positive mood have the potential to boost the development of these novel associations. The pink elephant introduced in the VR environment is an example of elements that patients might find helpful in overcoming food anxiety. This is corroborated by patients' qualitative feedback, which indicates that the pink elephant was perceived as a positive presence in the environment. It is interesting, however, that self‐reported levels of positive mood did not significantly increase more in the positive mood condition compared to the other two conditions. This raises the question as to whether improved positive mood is the putative mechanism explaining lower food anxiety. All patients provided qualitative feedback following the exposure to the virtual environment and those exposed to the pet, described it as “safe,” “helpful,” “non‐judgmental,” and “reassuring”. It is therefore possible that the pet might have induced feelings of safeness and reassurance rather than positive mood. This hypothesis warrants further investigation asking patients to rate more than one emotion after exposure to the pet. Also, the finding that positive mood induction might be associated with greater reduction in food‐related anxiety needs to be replicated and expanded in multiple exposure contexts (including exposure to food in real life) and using other measures of anxiety (e.g., through the registration of physiological parameters in response to food stimuli).

While exposure to the positive mood condition was associated with greater food‐related anxiety compared to the kitchen alone condition, it was somehow surprising that the same did not hold true for the social support condition. Although patients overall endorsed the idea to receive some kind of social support in the kitchen (e.g., “it's quite nice…to have that because there's that safe person”) and found the script supportive (e.g., “I thought that was very helpful. So that did make me a little bit emotional. It's helpful to hear reassurance while you're experiencing that scary thing”), they also tended to find the appearance of the avatar aversive (e.g., “also didn't really like the look of any of the avatars. They all looked a bit scary”; “Sometimes it was a bit scary because, like, you would just, like, turn around and she'd be there, like, right close”). Also, some commented that the experience of listening to the avatar while exploring the kitchen potentially was a bit overwhelming (e.g., “Like a bit too much. It was just a bit overwhelming…I was like…I'm just picking up a banana please leave me alone…!”). Future iterations of the visual appearance of the avatar and the recited script will need to consider these comments to develop more compassionate looking avatars and to enable patients to process the content of the script at a different timing (e.g., just before the exploration of the kitchen).

A secondary aim of this study was to compare the number of food touches and gazes toward virtual food items between conditions. No differences between food‐directed gazes were detected, but patients handled foods more often in the baseline condition, compared to the social support and positive mood conditions. Interestingly, the number of food touches was not associated with the reduction in food‐related anxiety found in the positive mood condition. This suggests that food “avoidance” (i.e., fewer food touches) did not explain the reduction in food anxiety. However, participants did not receive specific instructions with regards to which items to look at or touch. It is therefore difficult to interpret these data based on the lack of explicit instructions.

Overall, this study provides helpful information with regards to the feasibility of strategies to how to enhance inhibitory learning of food‐related fears in anorexia nervosa, in the context of exposure to virtual foods. The duration of virtual food exposure in this study was kept to a minimum, to test the feasibility of the approach. The next step is to examine the impact of multiple and longer virtual food exposure sessions on self‐reported food‐related anxiety, as well as anxiety at mealtimes and other indexes of fear reactivity, such as physiological parameters. Furthermore, considering that food fears and avoidance are transdiagnostic characteristics of eating disorders (Melles & Jansen, 2023), and that these are highest in anorexia nervosa and bulimia nervosa (Butler et al., 2023), it will be important to test similar protocols in other groups of patients, including those with bulimia nervosa or avoidant restrictive food intake disorder.

To conclude, the findings of this study indicate that using VR exposure to food stimuli enhanced by techniques for positive mood induction is feasible and associated with greater reduction of food‐related anxiety compared to the other two conditions, in a large sample of patients with anorexia nervosa. This study did not aim at delivering food exposure and testing its efficacy. The use of strategies to induce positive affect warrants further investigation in multiple‐session exposure studies, to replicate the potential impact on food‐related anxiety, both in the virtual environment and in vivo.

### 4.1 Limitations and strengths

This study has some limitations. The first one is that only one question was asked to measure the psychological states thought to be manipulated (e.g., positive mood and social support). The limited number of questions that were asked do not allow to establish whether more specific emotional states might have been induced by the pet or the avatar (e.g., feelings of connection, reassurance, self‐compassion). The second limitation, similarly, is that only a limited number of strategies were tested during food exposure. For example, patients were not asked to formulate their expectations prior to exposure and they were not exposed to different kinds of kitchen environments. Both limitations appear as such considering the number of questions that remain unanswered and the complexity of processes manipulated during treatment. At the same time, they have been driven by the choice to conduct an experimental study which enables controlling only a limited number of variables at the same time. More experimental studies are needed to test specific hypotheses with regards to which psychological strategy or which combination of psychological strategies work best to reduce negative emotions around food. Although this study might raise more questions than it answers, it represents an effort to isolate psychological strategies as they might happen during therapy, in the view of maximizing those which work.

A less general concern is that both the pet and the avatar might have worked as safety behaviors. Although the general indication is that safety behaviors (e.g., distraction) could hamper the efficacy of exposure, in this study, the pet and the avatar were aimed at creating a more positive emotional environment and therefore facilitate the development of new, “safer” learning about food. This raises an interesting reflection as to what clinicians should consider as unhelpful “safety behaviors” and what they should consider, instead as “safer behaviors,” which can provide confidence and motivation to patients to carry on engaging in difficult and uncomfortable experiences, such as food exposure.

This study did not assess the sense of presence in the virtual environment. Also, it did not include an assessment of in‐vivo eating behavior. Some of the qualitative feedback seemed to indicate that the virtual environment was “realistic” enough, while also enabling individuals to feel “safer” than at home. Also, some patients mentioned the potential for the virtual exposure to help with mealtimes at home. Future studies should test these hypotheses by measuring feelings of presence in the virtual environment and also potential changes in emotion reactivity at mealtimes following virtual food exposure. Finally, participants' BMI in the UK sample was self‐reported.

## AUTHOR CONTRIBUTIONS

**Ludovica Natali:** Data curation; formal analysis; investigation; visualization; writing – original draft; writing – review and editing. **Valentina Meregalli:** Data curation; investigation; writing – original draft; writing – review and editing. **Katie Paige Rowlands:** Data curation; investigation; writing – review and editing. **Jerome Di Pietro:** Software; writing – review and editing. **Janet Treasure:** Resources; writing – original draft; writing – review and editing. **enrico collantoni:** Writing – review and editing. **Paolo Meneguzzo:** Writing – review and editing. **Elena Tenconi:** Writing – review and editing. **Angela Favaro:** Resources; writing – review and editing. **Francesca Fontana:** Resources; writing – review and editing. **Enrico Ceccato:** Resources; writing – review and editing. **Alessandra Sala:** Resources; writing – review and editing. **Lucia Valmaggia:** Conceptualization; funding acquisition; methodology; project administration; supervision; writing – original draft; writing – review and editing. **Valentina Cardi:** Conceptualization; data curation; formal analysis; funding acquisition; investigation; methodology; project administration; resources; supervision; writing – original draft; writing – review and editing.

## CONFLICT OF INTEREST STATEMENT

The authors declare no conflicts of interest.

## ETHICS STATEMENT

The study received ethical approval from the ethical committees of the University of Padova (reference number: 4293) and hospital of Vicenza (reference number: 1831) in Italy, and from the Research Ethics Committee North West – Liverpool East (reference number: 18/NW/0853) in the UK. Patients and parents of those below 18 years of age provided written informed consent to participate in this study.

## Supporting information


**Table S1.** Participants' demographic and clinical variables expressed as means (standard deviations) or frequencies (%) for each virtual reality condition.

## Data Availability

The database is available upon request to the corresponding author.
