# Supplementary material for: Virtual food exposure with positive mood induction or social support to reduce food anxiety in anorexia nervosa: A feasibility study
Source: Int J Eat Disord. 2024 Feb 17;57(3):703–15. doi: 10.1002/eat.24155 (PMC11137760; doi:10.1002/eat.24155)
Supplement: Supplementary file 1 — Table S1. Participants' demographic and clinical variables expressed as means (standard deviations) or frequencies (%) for each virtual reality condition. [file EAT-57-703-s001.docx]

**Table S1**

*Participants’ demographic and clinical variables expressed as means (standard deviations) or frequencies (%) for each virtual reality condition.*

| **Variables** | **Virtual kitchen** | | **Virtual kitchen + social support** | | **Virtual kitchen + positive mood** | |
| --- | --- | --- | --- | --- | --- | --- |
|  | ***N*** | ***M (SD)* or Frequency (%)** | ***N*** | ***M (SD)* or Frequency (%)** | ***N*** | ***M (SD)* or Frequency (%)** |
| Age | 49 | 22.61(7.84) | 46 | 21.37(5.33) | 49 | 21.25(6.60) |
| Gender | 50 |  | 46 |  | 49 |  |
| Female |  | 47(94.00%) |  | 44(95.65%) |  | 49(100%) |
| Male |  | 1(2.00%) |  | 1(2.17%) |  | 0(0.00%) |
| Non-binary |  | 2(4.00%) |  | 0(0.00%) |  | 0(0.00%) |
| Other |  | 0(0.00%) |  | 1(2.17%) |  | 0(0.00%) |
| Data collection site (Italy vs. UK) | 50 | 31(62.00%) | 46 | 30(65.22%) | 49 | 30(61.22%) |
| Ethnicity (White vs. Other) | 50 | 46(92.00%) | 45 | 44(95.65%) | 49 | 46(93.87%) |
| Illness Duration (Years) | 49 | 6.28(6.03) | 45 | 5.09(4.69) | 48 | 4.99(6.15) |
| Years of education | 50 | 13.62(2.93) | 46 | 13.59(3.54) | 49 | 13.08(3.48) |
| Body Mass Index (Kg/m^2^)^a^ | 32 | 16.34(2.02) | 27 | 18.20(2.71) | 28 | 16.59(2.64) |
| Percent median BMI^b^ | 12 | 81.60(8.04) | 12 | 83.53(6.25) | 17 | 84.13(9.72) |
| Binge eating (Yes vs No)^c^ | 50 | 6(12.00%) | 46 | 10(21.74%) | 49 | 7(14.29%) |
| Purging (Yes vs No)^d^ | 50 | 8(16.00%) | 46 | 12(26.09%) | 49 | 8(16.33%) |
| EDE-Q Global Score | 50 | 4.26(0.80) | 46 | 4.41(0.75) | 49 | 4.19(0.75) |
| EDE-Q Restraint | 50 | 4.01(1.21) | 46 | 4.12(1.22) | 49 | 3.96(1.16) |
| EDE-Q Eating Concern | 50 | 3.50(1.16) | 46 | 3.66(0.98) | 49 | 3.27(0.99) |
| EDE-Q Weight Concern | 50 | 4.58(1.06) | 46 | 4.67(1.07) | 49 | 4.54(1.07) |
| EDE-Q Shape Concern | 50 | 4.96(0.85) | 46 | 5.20(0.78) | 49 | 4.97(0.83) |
| DASS Total Score | 50 | 72.32(22.56) | 46 | 70.87(23.27) | 49 | 63.02(23.73) |

*Note*. Socio-demographic and clinical variables expressed as mean (standard deviation), and frequency (percentage).

^a^Calculated for patients aged 18 or older.

^b^Calculated only for underage patients.

^c^ “Binge eating” is a categorial variable: a “yes” was given when participants reported at least four binge episodes in the last month.

^d^“Purging” is a categorial variable: a “yes” was given when participants reported at least one episode in the last month.
